# Ethanolic Extract of *Mangifera indica* Protects against CCl_4_-Induced Hepatotoxicity via Antioxidant Capabilities in Albino Rats

**DOI:** 10.1155/2024/5539386

**Published:** 2024-08-27

**Authors:** Ahmed Saeed Kabbashi, Salwa Abdulla Eltawaty, Amar Mohamed Ismail, Ahmed Abdelhhafiz Elshikh, Ayat Ahmed Alrasheid, Rawan Ahmed Elmahi, Waleed S. Koko, Elbadri Elamin Osman

**Affiliations:** ^1^ Department of Biomedical Science Faculty of Pharmacy Omar Al-Mukhtar University, Al-Bayda, Libya; ^2^ Department of Botany Faculty of Science and Technology Omdurman Islamic University, Omdurman, Sudan; ^3^ Department of Pharmacognosy Faculty of Pharmacy University of Medical Sciences and Technology, Khartoum, Sudan; ^4^ Department of Histopathology Faculty of Medical Laboratory Sciences International University of Africa, Khartoum, Sudan; ^5^ Department of Biology College of Science Qassim University, Qassim 51452, Saudi Arabia; ^6^ Department of Microbiology Faculty of Pure and Applied Science International University of Africa, Khartoum, Sudan

## Abstract

**Objective:**

To investigate the antioxidant and hepatoprotective effects of ethanolic *Mangifera indica* (*M. indica*) seed extract on carbon tetrachloride (CCl_4_)-induced hepatotoxicity in albino rats.

**Methods:**

Forty-eight albino rats weighing (100–150 g) were used for hepatoprotective and toxicity experiments. Antioxidant activity was determined using the 2, 2-diphenyl-1-picrylhydrazyl (DPPH) assay. The toxicity of *M. indica* seeds on the liver was evaluated by examining wellness parameters, body weight, and liver histological sections. The protective effects of 50 mg/kg and 100 mg/kg of seed extract on CCl_4_-induced hepatotoxicity were investigated by evaluating hematological, renal, and liver function parameters, body weight, and liver histological sections.

**Results:**

The antioxidant activity of the *M. indica* ethanolic extract was (92 ± 0.03 RSA %) compared with (91 ± 0.01 RSA %) of propyl gallate, and the IC_50_ was (8.3 ± 0.01 *µ*g/ml) and (14.1 ± 0.01 *µ*g/ml). No changes were observed in the health indicators, body weights, and liver histological sections following oral administration of 50 mg/kg and 100 mg/kg of *M. indica* seed extracts. Treatment with *M. indica* seed extract significantly reduced alanine transaminase (ALT), aspartate transaminase (AST), alkaline phosphatase (ALP), blood sugar, and urea levels compared with those in the CCl_4_-treated group.

**Conclusion:**

The IC_50_ of the *M. indica* ethanolic extract was 8.3 *µ*g/ml, and the *M. indica* extract is a potential source of natural antioxidants that protect against CCl_4_-induced hepatotoxicity.

## 1. Introduction

Herbal remedies often include plant antioxidants such as polyphenols, flavonoids, and phenolic compounds, which act as free radical scavengers to protect against damage caused by oxidative stress [1]. The liver is the initial line of defense in the body's protective system as it detoxifies and eliminates toxic and foreign substances [2, 3]. Previous studies have shown that oxidative stress, inflammation, and toxic compounds contribute to hepatocellular damage [4]. In experimental models, CCl_4_ is commonly used to induce hepatocellular damage and promote hepatotoxicity [5, 6], leading to liver enzyme leakage [7]. Antioxidants can help reduce the risk of liver disorders by preventing oxidative damage caused by free radicals such as trichloromethyl free radicals and reactive oxygen species (ROS) generated by CCl_4_. Herbal remedies have been used for centuries to treat various illnesses [8]. Natural products are an important source of many medications, including anticancer drugs, anti-inflammatory agents, antioxidants, and detoxifying agents [9]. *M. indica* referred to as mango is a plant in the Anacardiaceae family widely used in pharmacology, ethnomedicine, and phytochemistry. The *M. indica* tree is used in traditional medicine to treat various diseases, and it contains polyphenols, terpenes, sterols, carotenoids, vitamins, and amino acids [10]. Several studies have demonstrated the pharmacological effects of the leaves, bark, fruit peel and flesh, roots, and flowers of mango trees [11], hepatoprotective [12], radioprotective [13], cell migration [14], antidiarrheal [15], anticancer [16], and antimicrobial activities [17]. The primary components of the pulp include water, carbohydrates, organic acids, lipids, minerals, colors, tannins, vitamins, and flavoring compounds, and it also contains large amounts of vitamins A, C, and D. According to [18], the ester and carbonyl types constitute the characteristic scent that evolves throughout the ripening process. However, the possible antioxidant and hepatoprotective properties of *M. indica* seed extracts have been poorly studied, and the large number of discarded mango seeds encourages their use because of their high productivity and low cost. Therefore, this study was conducted to investigate the antioxidant and hepatoprotective effects of the ethanolic extract of *M. indica* seeds on CCl_4_-induced hepatotoxicity in rats.

## 2. Materials and Methods

### 2.1. Chemicals and Reagents

Oxford Lab Fine Chem LLP (India) sells carbon tetrachloride (CCl_4_). Liquid paraffin (Oxford Lab Fine Chem LLP, India), 10% formalin (Novochem Engineering, India), the standard drug silymarin (Sigma-Aldrich), ethanol (SIGMA-ALDRICH, Germany), xylene (Scien TEST-bioKEMIX GmbH, Germany), kits for liver chemistry (Humana, Germany), DPPH (Chemos GmbH and Co.KG. Germany), and hematoxylin and eosin (H&E) (Santa Cruz Biotechnology, Inc., USA).

### 2.2. Collection of Plant Materials

Fresh and high-quality *M. indica* seeds were collected from Khartoum State between January and May 2019. The *M. indica* was accurately identified and confirmed by the taxonomists at the Herbarium of Medicinal and Aromatic Plants and Traditional Medicine Research Institute (MAPTMRI) in Khartoum, Sudan. The seeds were air-dried in a well-ventilated area in the shade, and the powder was prepared for extraction.

### 2.3. Preparation of Crude Extract

*M. indica* seeds were prepared using an overnight maceration process [19], and 50 g of crushed seeds were macerated in 500 mL of 80% ethanol for three days at room temperature. The supernatant was removed by random shaking for 24 hours at room temperature. The supernatant was filtered at 55°C by rotary evaporation at a low pressure. Each residue was weighed, and the yield percentage was calculated. The glass vial containing the extract was carefully sealed and stored at 4°C. After drying, the extract was stored in a deep freezer (Virtis, USA) for 48 h before use.

### 2.4. Antioxidant Activity (DPPH Assay) of the *M. indica* Extract

The antioxidant activity was evaluated using the stable radical, 2.2 di-(4-tretoctylphenyl)-1-picrylhydrazyl stable free radical (DPPH), which is the ability to donate hydrogen or scavenge free radicals, with minor modifications as previously described [20]. The DPPH radical was converted to purple diphenyl-picrylhydrazine. The extracts and standards were then added to the appropriate microplate wells containing DPPH and incubated at 37°C for 30 min while maintaining the level of DPPH at 300 mM. After incubation, the reduction in the absorbance at 517 nm was measured using an ELISA reader spectrophotometer. The percentage of antioxidant activity was calculated using the following formula:

(1) $$\begin{array}{c} \text{DPPH radical scavenging }\left(\%\right)=100-\left\{\frac{\left(\text{Ac}-\text{At}\right)}{\text{Ac}}\right\}\times 100, \end{array}$$

where At = absorbance value of the test compound; Ac = absorbance value of the control.

The IC_50_ of the extract was calculated using the EZ-Fit Enzyme Kinetic Program (Perrella Scientific Inc, U.S.A).

### 2.5. Experimental Animals and *M. indica* Extract Toxicity

Albino rats weighing 100–150 g were used to assess the subchronic toxicity of the *M. indica* extract. The rats were housed in an environment with a humidity of 40-50%, a 12-hour light/dark cycle, and a room temperature of 22–24°C for 14 days. The rats were divided randomly into three groups of six rats each. Group 1 untreated group served as the control group, Group 2 received 50 mg/kg/day of *M. indica* extract, and Group 3 was administered 100 mg/kg/day for 14 days. The rats were fasted overnight for 18 hours. After anesthesia, rats were sacrificed and clinical signs were recorded. Blood samples were collected, and serum was obtained after centrifugation at 3000 rpm. All animal experiments were performed following the principles of the Declaration of Helsinki.

### 2.6. Estimation of Biochemical Parameters

Serum AST, ALT, and ALP activities were determined using kinetic methods, while protein, urea, albumin, and calcium levels were evaluated using a fully automated chemistry analyzer. Tissue specimens from the liver, kidneys, heart, spleen, and brain were carefully removed, weighed, fixed in 10% formal saline, and prepared for histopathological analyses. The relative organ weight of each animal was calculated using the following formula:

(2) $$\begin{array}{c} \text{Relative organ weight}=\frac{\left(\text{organ weight }\left(g\right)\right)}{\left(\text{body weight of the animal on sacrifice day }\left(g\right)\right)}\times 100. \end{array}$$

Visual observations of mortality and changes in physical appearance and behavior (sleepiness, salivation, and lethargy) were recorded.

### 2.7. Experimental Animals and *M. indica* Extract Hepatoprotective Activity

This study aimed to evaluate hepatotoxicity induced by CCl_4_-induced liver injury in rats. Thirty adult albino rats weighing 100–150 g were divided into five groups of six rats each. Group 1 was treated with olive oil 0.2 ml/kg/3 times a week for 10 days. Group 2 was treated intraperitoneally with CCl_4_ 0.2 ml/kg dissolved in equal volume olive oil (V/V). Group 3 was treated with 100 mg/kg *M. indica* extract and 0.2 ml/kg/day of CCl_4_. Group 4 received 50 mg/kg of the extract and 0.2 ml/kg 3 times a week for 10 days. Group 5 received CCl_4_ 0.2 ml/kg and silymarin suspended in 5% acacia mucilage. After ten days, the rats were fasted and euthanized and blood samples were collected. Serum was obtained after centrifugation at 3000 rpm. A fully automated analyzer was used to measure the biochemical parameters. The livers were removed, fixed in 10% formal saline, embedded in paraffin wax, sectioned at 5 *µ*m thickness, and stained with H & E using Mayer's hemalum. All experiments were conducted in accordance with the Declaration of Helsinki.

### 2.8. Statistical Analysis

Statistical Package for (SPSS) software (version 21.0 (SPSS ver. 21.0, Inc., Chicago, IL, USA) was used for the statistical analysis. Mean ± standard error of the mean (SEM) and percentage (%) were used to represent the data. Independent *t*-tests and analysis of variance (ANOVA) were used to compare group means. Differences were considered statistically significant at a *p* value of 0.05.

## 3. Results

### 3.1. Antioxidant Activity of *M. indica* Extract

The results of DPPH radical scavenging activity show that the *M. indica* extract had a higher antioxidant activity (92 ± 0.03 RSA% than propyl gallate (91 ± 0.01 RSA%) and the lowest IC_50_ (8.3 ± 0.01 *µ*g/ml) and (14.1 ± 0.01 *µ*g/ml), respectively, presented in Table 1.

### 3.2. The Effect of *M. indica* Extract on Clinical Signs and Behaviors

The acute oral toxicity of the ethanolic extract of *M. indica* was examined after 14 days. As shown in Table 2, no toxic symptoms were observed and signs of depression and mortality were reported throughout the study period. Additionally, no treatment-related flaws or overt clinical indications of toxicity, stress, or changes in appearance or behavior were observed. Moreover, the liver histological analysis of the extract-treated and control groups revealed no significant differences.

### 3.3. The Effect of *M*. *indica* Extract on Body Weight

**Figure 1:**
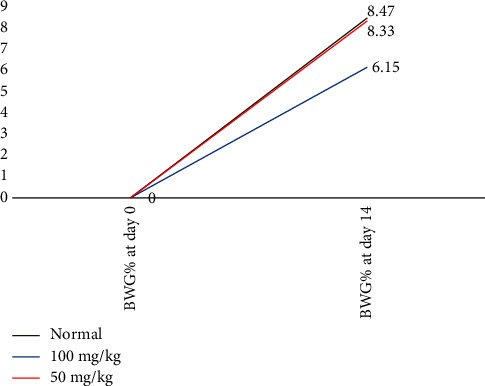
Comparison of body weight gain percentage (BWG %) in study groups.

As shown in Table 3 and Figure 1, the body weight gain of the group treated with *M. indica* extract 100 mg/kg was significantly decreased (6.15%) compared to the control group (8.47%), and in contrast, no change was observed in the group treated with 50 mg/kg (8.33%).

### 3.4. Hepatoprotective Activity of *M. indica* Extract

**Figure 2:**
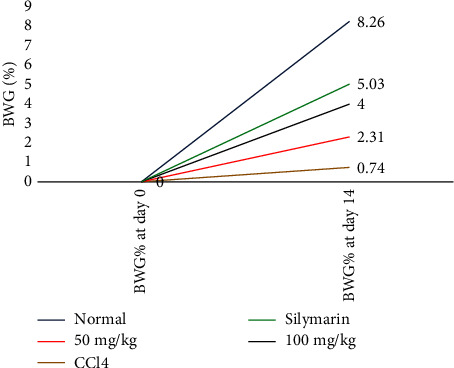
Effect of *M. indica* extract on body weight gain (%) in study groups.

Compared to the baseline, CCl_4_ therapy caused a substantial increase in liver size (2.10 g) and reduced BWG % (0.74%); in contrast, treatment with 100 mg/kg and low 50 mg/kg extracts resulted in BWG % reduction (4.00% and 2.31%) and no change in liver size (1.65 g and 1.68 g), respectively, as indicated in Table 4 and Figure 2.

### 3.5. The Effect of *M. indica* Extract on Biochemical Markers

The biochemical profile was investigated using a fully automated chemistry analyzer after treatment with 50 and 100 mg/kg/day for 10 days. Treatment with 100 mg/kg of *M. indica* extract significantly reduced ALT (130 ± 1.37 U/l), AST (149 ± 2.20 U/l), ALP (141 ± 1.86 U/l) activities, blood glucose levels (74.1 ± 1.10 mg/dl), and blood urea level (35.9 ± 3.05 mg/dl) when compared with the CCl_4_-induced liver cells damage group ALT (153 ± 0.76 U/l), AST (165 ± 2.24 U/l), ALP (187 ± 3.81 U/l), and blood glucose (95.0 ± 1.09 mg/dl) and 61.2 ± 0.09 mg/dl), respectively. In contrast, silymarin significantly decreased the activities of ALT (120 ± 0.71 U/l), AST (142 ± 2.62 U/l), ALP (128 ± 2.10 U/l), blood glucose levels (73.2 ± 1.05 mg/dl), and urea (36.4 ± 1.23 mg/dl), respectively, are presented in Table 5.

### 3.6. Histopathological Findings of *M. indica* Extract

**Figure 3:**
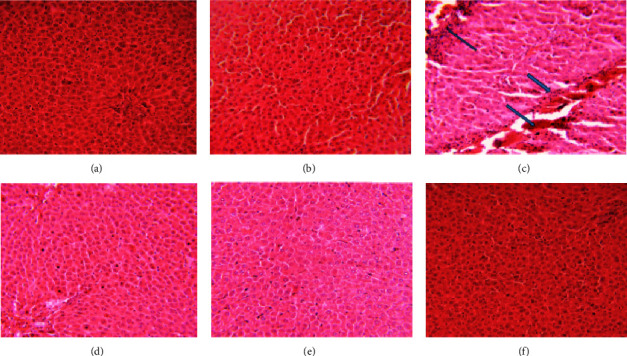
Microscopic examination of liver tissue morphology: (a) liquid paraffin control-treated group exhibits normal hepatocyte, (b) silymarin (10 mg/kg) as hepatoprotective drug control, (c) *M. indica* extract (50 mg/kg) combined with CCl_4_ exhibits recovery of normal hepatocyte, (d) *M. indica* extract (100 mg/kg) induced with CCl_4_ shows the significant recovery of normal hepatocyte, (e) CCl_4_ (0.2 mg/kg) induced toxicity, and (f) treatment with *M. indica* extract alone (100 mg/kg).

To assess the possible protective effects of *M. indica* on liver cells damage induced by CCL_4_, histopathological analysis of the liver tissue was performed. The results indicated that CCl_4_ treatment led to the emergence of fatty liver cells, necrosis, hyperplasia, infiltration, and inflammation, in contrast to the normal liver cells treated with both low and high doses of *M. indica* (Figures 3(a), 3(b), 3(c), 3(d), 3(e), and 3(f)).

## 4. Discussion

In tropical regions, *M. indica* has traditionally been used as a medicinal plant, and scientific studies have confirmed its positive effects on health [21]. This study aimed to investigate the antioxidant and hepatoprotective properties of *M. indica* ethanolic seed extracts against CCl_4_-induced liver damage in rats. This study revealed that the *M. indica* extract protects against CCl_4_-induced hepatotoxicity and is nonharmful. The protective effects of the extracts are attributed to their antioxidant components, such as phenolics, which protect cells from damage [22, 23]. In line with some studies, our results indicated that the ethanolic extract of *M. indica* could serve as a natural source of antioxidants that protect against CCl_4_-induced cell damage.

The DPPH assay was performed to assess the antioxidant properties of *M. indica* ethanolic extract. The results demonstrated that the ethanolic extract of *M. indica* exhibited the highest radical scavenging capacity and the lowest IC_50_ value compared with propyl gallate. These results align with those of previous studies, which have found that the polyphenols, carotenoids, and tocopherols of *M. indica* seeds neutralize free radicals, thereby protecting against oxidative stress and the development of diseases, such as cancer, Alzheimer's disease, and inflammation [24, 25].

In this study, the in vitro cytotoxic activities of *M. indica* seed methanolic extracts were evaluated and compared to those of the control. Following a 14-day administration of the *M. indica* ethanolic seed extract, clinical symptoms, behavior, wellness, and body weights were evaluated. The study revealed no noticeable discrepancies between the groups that received *M. indica* and the control for any of the examined aspects. These data confirm the safety of this extract. Previous research has also documented a lack of toxic reactions to the stem bark of *M. indica* in albino rats. However, high-dose treatment with *M. indica* was found to lead to reduced body weight, which may explain its delayed use in severe illness, weight loss, and improved quality of life in Cuba [26].

The *M. indica* extract exhibited efficacy in safeguarding against CCl_4_-induced hepatotoxicity, as evidenced by the notable decreases in ALT, AST, ALP activities, blood glucose, and blood urea levels. Furthermore, the normal hepatocellular histology observed in the extract-treated group compared to that in the CCl_4_-treated group indicated the effectiveness of the *M. indica* extract. Elevated transaminase activity indicates that CCl_4_ triggers the release of ROS, which causes hepatocellular damage, and higher ALP activity suggests hepatobiliary damage. These findings were further confirmed by the presence of fatty liver and necrosis in the liver tissues of the CCl_4_-treated group. Increased blood urea levels suggested impaired renal function. The low blood glucose levels observed after the administration of the extract may be attributed to the antioxidant activity of *M. indica*, which protects against *β*-cell damage induced by CCl_4_. Several plant products have been shown to protect against and minimize hepatocellular damage caused by the chemical agent [27]. Herbal medicine has recently received much attention as a complementary diet and adjuvant therapy for preventing and treating a wide range of diseases such as cancer, cardiovascular diseases, and Alzheimer's disease. This suggests that *M. indica* exhibits significant antioxidant effects against chemicals and oxidative stress-induced cellular damage. A previous study on *M. indica* leaf extracts demonstrated that all parts of *M. indica* possess considerable antioxidant activity, similar to the leaves [28].

The effects of the *M. indica* extract on liver histological sections were examined. Histological sections of the group treated with *M. indica* seed extract showed hepatoprotective effects. The results indicated that the CCl_4_-treated group exhibited fatty liver, necrosis, hyperplasia, infiltration, and inflammation, while the liver cells in the *M. indica*-treated groups appeared healthy. Previous studies on albino rats found that *M. indica* leaves had hepatoprotective properties against CCl_4_-induced liver injury [29, 30]. However, one study reported that the administration of aqueous and ethanolic extracts of *M. indica* stem bark significantly increases AST and ALT activities [31]. The limitations of this study include the exclusion of ammonia, acute inflammatory markers, and antioxidant enzymes, which provide insights into the protective mechanisms of *M. indica* against hepatocellular damage, and the lack of clinical data.

## 5. Conclusion

In this study, we evaluated the antioxidant and hepatoprotective effects of the *M. indica* seed extract against CCl_4_-induced toxicity. *M. indica* exhibits antioxidant activity and protects against CCl_4_-induced hepatotoxicity, with an IC_50_ of 8.3 *µ*g/ml. Treatment with the *M. indica* seed extract decreased the levels of transaminases, ALP, urea, and blood glucose, thereby protecting against CCl_4_-induced hepatocellular damage. Based on these findings, *M. indica* appears to be a safe, effective, and promising natural product for use in herbal medicine. Further research is warranted to elucidate the underlying mechanisms by which *M. indica* protects against cellular damage.

## Figures and Tables

**Table 1:** Percentage radical scavenging activity (%RSA) and IC_50_ (mg/ml) of *M. indica* extract using DPPH assay.

Treatments	Solvent	%RSA^∗^ ± SD (DPPH)	IC_50_ ± SD (*µ*g/ml)
*Mangifera indica*	Ethanolic	92 ± 0.03	8.3 ± 0.01
Propyl gallate	Std	91 ± 0.01	14.1 ± 0.01

Key: RSA^∗^: radical scavenging activity, (*n*; 3), DPPH: 2, 2, diphenyl-1-picrylhydrazyl, SD: standard division, Std.: standard. IC_50_: value represents the concentration of the sample required to inhibit 50% of DPPH free radical.

**Table 2:** The effect of *M. indica* extract on wellness parameters in study groups.

Name of plant	Observations	Animals/concentrations		
		G_1_/Normal	G_2_/50 mg/kg	G_3_/100 mg/kg
*M. indica*	Eyes	Normal	Normal	Normal
	Mucous membrane	Normal	Normal	Normal
	Salivation	Normal	Normal	Normal
	Skin and fur	Normal	Normal	Normal
	Lethargy	Absent	Absent	Absent
	Sleep	Normal	Normal	Normal
	Coma	Absent	Absent	Absent
	Convulsion	Absent	Absent	Absent
	Tremors	Absent	Absent	Absent
	Diarrhea	Absent	Absent	Absent
	Mortality	Nil	Nil	Nil

**Table 3:** The effect of *M. indica* on body weight in study groups.

Groups	Body weight (gm)		Change in b. wt.	b. wt. gain or loss (%)	b. wt. gain or loss
	Initial	Final			
Normal	118 ± 0.20	128 ± 1.05	10.0	8.47	Gain
100 mg/kg	130 ± 0.59	138 ± 0.90	08.0	6.15	Gain
50 mg/kg	120 ± 1.80	130 ± 0.10	10.0	8.33	Gain

**Table 4:** Effect of *M. indica* extract on body weight, change in body weight (g), body weight, and relative liver weight in CCl_4_-induced hepatotoxicity.

Groups	Body weight (gm)		Change in b. wt.	b. wt. gain or loss (%)	b. wt. gain or loss	Liver weight (gm)	Relative liver weight (%)
	Initial	Final					
Normal	121 ± 1.20	131 ± 2.00	10.0	8.26	Gain	1.60	1.22 ± 0.11
CCl_4_	135 ± 2.00	136 ± 1.70	01.0	0.74	Gain	2.10	1.45 ± 0.50
100 ml/kg	125 ± 1.50	130 ± 0.90	05.0	4.00	Gain	1.65	1.26 ± 0.20
50 ml/kg	130 ± 3.60	133 ± 3.10	03.0	2.31	Gain	1.82	1.36 ± 0.30
Silymarin	139 ± 2.90	146 ± 2.25	07.0	5.03	Gain	1.65	1.13 ± 0.10

Key: each value represents the mean ± standard error of mean (mean ± SEM).

**Table 5:** Comparison of biochemical parameters (mean ± SEM) in study groups.

Rats group biochemical parameters	Normal	CCl_4_	1 ml/kg	0.5 ml/kg	Silymarin
	Mean ± SEM				
Urea (mg/dl)	28.2 ± 0.12	61.2 ± 0.09	40.7 ± 1.07	35.9 ± 1.05	36.4 ± 1.23
Glucose (mg/dl)	62.1 ± 0.06	95.0 ± 1.09	74.1 ± 1.10	76.4 ± 1.33	73.2 ± 1.05
Creatinine (mg/dl)	0.79 ± 0.05	0.94 ± 0.07	0.90 ± 0.10	0.79 ± 0.04	0.80 ± 0.06
Albumin (mg/dl)	3.50 ± 0.07	5.80 ± 1.09	4.50 ± 1.00	3.50 ± 0.96	3.60 ± 0.26
Total protein (mg/dl)	8.29 ± 0.07	9.98 ± 0.04	8.18 ± 0.11	8.58 ± 0.10	8.21 ± 0.07
ALT (U/L)	122 ± 2.65	153 ± 0.76	130 ± 1.37	140 ± 1.28	120 ± 0.71
AST (U/L)	130 ± 2.69	165 ± 2.24	149 ± 2.20	148 ± 1.61	142 ± 2.62
ALP (U/L)	137 ± 1.81	187 ± 3.81	141 ± 1.86	131 ± 4.54	128 ± 2.10

Key: values expressed as a mean ± standard error of mean (SEM).

## Data Availability

The data used to support the findings of this study are available from the corresponding author upon request.

## Abbreviations

ALP: Alkaline phosphatase
ALT: Alanine aminotransferase
AST: Aspartate aminotransferase
CCl_4_: Carbon tetrachloride
DPPH: 2,2-diphenyl-1-picrylhydrazyl
IC_50_: Half maximal inhibitory concentration
*M. indica*: *Mangifera indica*
MAPTMRI: Medicinal and aromatic plants and traditional medicine research institute
ROS: Reactive oxygen species.
